# A promising platform for predicting toxicity

**DOI:** 10.7554/eLife.73191

**Published:** 2021-09-24

**Authors:** Maria Ochoa de Olza

**Affiliations:** Lausanne University Hospital Lausanne Switzerland

**Keywords:** organs-on-chips, t-cell bispecifics, cancer immunotherapy, preclinical safety, alveolar biology, intestinal biology, Human

## Abstract

Organ-on-chip approaches could help researchers to better predict the toxicity of cancer immunotherapy drugs.

**Related research article** Kerns SJ, Belgur C, Petropolis D, Kanellias M, Barrile R, Sam J, Weinzierl T, Fauti T, Freimoser-Grundschober A, Eckmann J, Hage C, Geiger M, Ng PR, Tien-Street W, Manatakis DV, Micallef V, Gerard R, Bscheider M, Breous-Nystrom E, Schneider A, Giusti AM, Bertinetti-Lapatki C, Grant HS, Roth AB, Hamilton GA, Singer T, Karalis K, Moisan A, Bruenker P, Klein C, Bacac M, Gjorevski N, Cabon L. 2021. Human immunocompetent Organ-on-Chip platforms allow safety profiling of tumor-targeted T-cell bispecific antibodies. *eLife* **10**:e67106. doi: 10.7554/eLife.67106

Killing cancer cells can be done directly or by harnessing the immune system through an approach called cancer immunotherapy (Robert, 2020). Indeed, over the past decade, most of the advances in oncology have involved boosting immune cells to destroy tumor cells. Some of these treatments have resulted in impressive improvements in survival, bringing the possibility of a cure closer for some patients (Robert et al., 2018). This is a big milestone in oncology.

Despite these advances, immunotherapies can be associated with toxicity, which forces the treatment to be stopped: if immune cells become too activated, they can mistakenly recognize and destroy healthy tissues. This can lead, for example, to rashes, hepatitis or colitis depending on whether the skin, liver or gut are attacked (Marin-Acevedo et al., 2019).

A big challenge in oncology is therefore to predict which new immunotherapy drugs are going to be too harmful for patients. This is usually examined in animal models, but since their immune systems differ from the human immune system, it can be difficult to reliably predict toxicity (Zschaler et al., 2014). Now, in eLife, Nikolce Gjorevski (Roche), Lauriane Cabon (Roche) and colleagues – including Jordan Kerns and Chaitra Belgur of Emulate Inc in Boston as joint first authors – report how an in vitro model can help bypass this problem for T cell bispecific antibodies immunotherapy (Kerns et al., 2021).

T cell bispecific antibodies (or TCBs) can recognise and bind to ‘antigen’ proteins present on the surface of tumors, as well as receptors displayed by immune ‘T cells’: by bringing the two types of cells closer, this process helps to activate T cells and allows them to kill their targets. However, the antigens that TCBs bind to are not always exclusive to cancer cells. Recognition of non-cancer cells which share antigens with tumors – known as the on-target, off-tumor effect – can lead to normal cells being damaged (Labrijn et al., 2019; Figure 1). Predicting which TCBs under clinical development will cause such undesired toxicity represents an important challenge in oncology.

**Figure 1.**
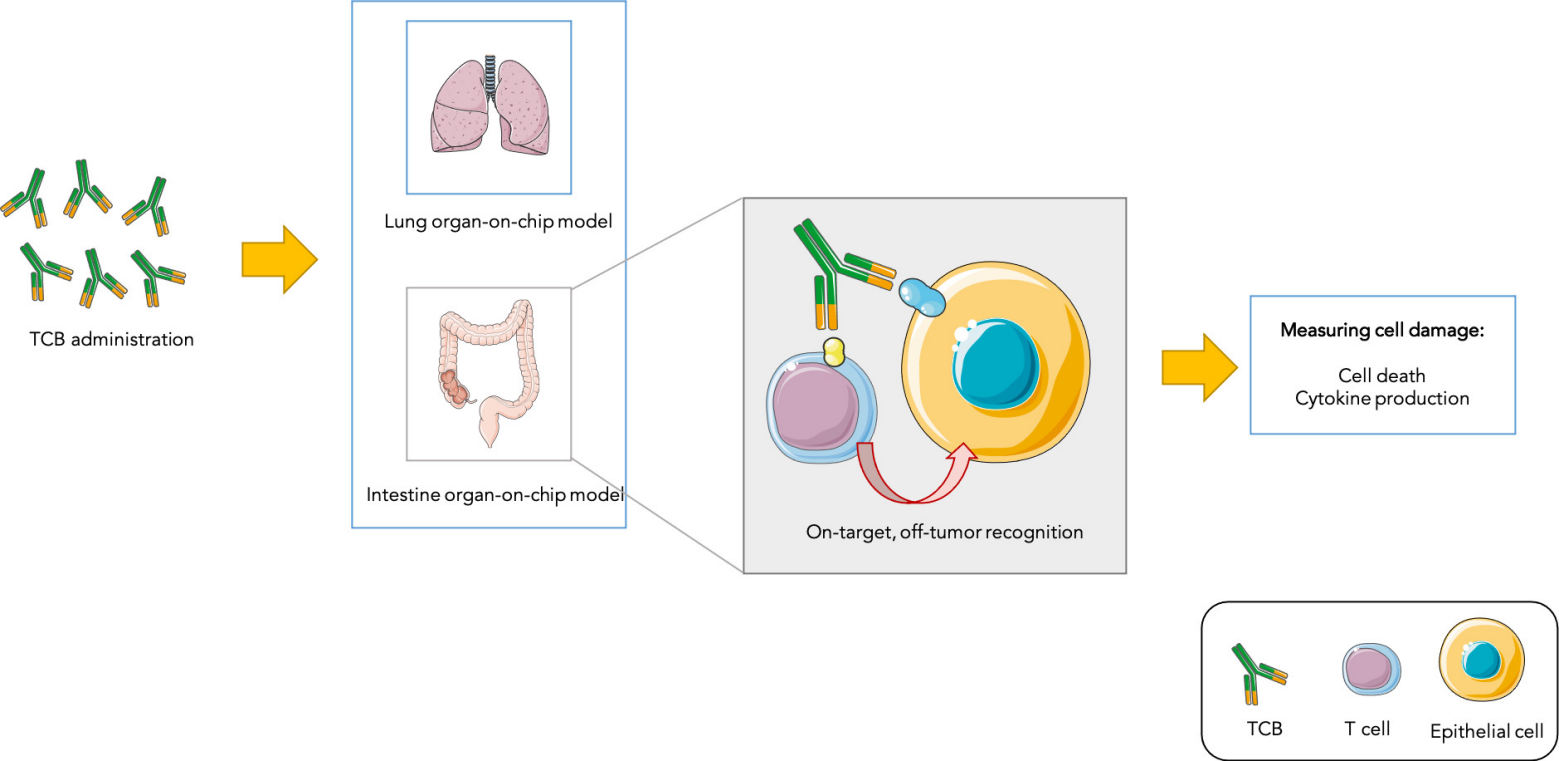
Testing drug toxicity using an organ-on-chip platform. Administering TCBs to an organ-on-chip platform (such as a model of the lung or intestine) allows the antibodies (Y-shaped structures) to recognize antigens (shown in yellow and blue) present in both healthy epithelial and cancerous cells. This brings T cells close to their targets and also activates them, which can lead to healthy cells being damaged (as assessed by measuring cell death and inflammation levels). This model is able to partially reproduce toxicity observed in animals and humans.

Image credit: Image was prepared with Servier Medical Art (CC BY 3.0).

To address this issue, Kerns et al. first took advantage of a lung-on-chip model (Huh et al., 2010) – a system grown under conditions mimicking those found in the body – to predict toxicity to TCBs. This ‘mini-organ’ was exposed to a TCB that recognises an antigen present in ovarian, lung and breast cancer cells, but which can also be expressed, at lower levels, in healthy lung cells. This manipulation led to healthy cells being damaged in the lung-on-chip model, which was then used to determine which TCB dose could kill tumors while sparing normal lung cells. This dose was then administered to mouse models, whose lungs remained undamaged. This demonstrates that the lung-on-chip was able to efficiently predict toxicity in these animals.

Kerns et al. then used an intestine-on-chip model to test a TCB which targets an antigen present on both colon cancer and normal intestine cells. Healthy cells survived the treatment but signs of toxicity emerged that could mimick side-effects that commonly occur in patients, such as increased inflammation.

Using an organ-on-chip model to test TCBs therefore has two main advantages. First, it reproduces toxicity observed in vivo, and can help to determine which doses are effective while remaining safe for normal cells. Second, it serves to predict toxicity before patients are administered with newly-developed TCBs, filling the gap between animal and human studies for drugs that are yet to be clinically tested. Organ-on-chip models could therefore make drug development more efficient by helping to screen out toxic TCBs before they reach cancer patients.
